# The impact of ondansetron on clinical outcomes in cranial surgery patients: a propensity-matched analysis of retrospective data

**DOI:** 10.3389/fnagi.2025.1627353

**Published:** 2025-09-23

**Authors:** Debo Yun, Yongzhen Luo, Rui Li, Wenyong Li, Chunyou Wan, Qinjiang Huang, Jiang Du, Shiyi Zeng, Guangdong Wang

**Affiliations:** ^1^Department of Neurosurgery, Chengdu Wenjiang District People’s Hospital, Chengdu, China; ^2^Department of Respiratory and Critical Care Medicine, First Affiliated Hospital of Xi’an Jiaotong University, Xi’an, China

**Keywords:** cranial surgery, ondansetron, mortality, propensity score matching, clinical outcomes

## Abstract

**Introduction:**

Cranial surgery represents a substantial public health challenge, characterized by intricate postoperative management that can complicate patient recovery. This study investigates the effect of ondansetron, a serotonin receptor antagonist, on clinical outcomes in adult patients undergoing cranial surgery, addressing the uncertainty surrounding its impact on postoperative complications.

**Methods:**

Utilizing a retrospective cohort design, we analyzed data from 2,297 eligible patients, segregating them into ondansetron-treated and control groups, while applying propensity score matching to harmonize baseline characteristics. The primary outcomes assessed were 28-day and 90-day mortality, evaluated using multivariable Cox regression and Kaplan-Meier analysis.

**Results:**

After matching, 905 well-balanced pairs were included. Ondansetron administration was associated with significantly lower 28-day mortality (hazard ratio [HR] = 0.69, 95% confidence interval [CI]: 0.52–0.92; *P* = 0.012) and 90-day mortality (HR = 0.74, 95% CI: 0.58–0.94; *P* = 0.014) after adjusting for potential confounders. Kaplan-Meier survival analysis further corroborated these findings, showing a consistent protective effect of ondansetron with significant mortality reduction at both 28 days (HR = 0.68, 95% CI: 0.51–0.91; *P* = 0.009) and 90 days (HR = 0.75, 95% CI: 0.59–0.95; *P* = 0.015), with subgroup analyses confirming result stability across demographic factors.

**Conclusion:**

The administration of ondansetron in cranial surgery patients with a Glasgow Coma Scale score exceeding eight is associated with a significant reduction in short-term mortality, suggesting that ondansetron could be a viable therapeutic strategy to enhance postoperative recovery outcomes.

## 1 Introduction

Cranial surgery serves as an essential therapeutic modality in neurosurgical practice for conditions including traumatic brain injury, cerebrovascular diseases, intracranial tumors, functional brain disorders, and intracranial infections, representing a critical intervention for these pathologies (Cagney et al., 2019; Ramakonar et al., 2018). Postoperative care is equally critical as surgical success, making brief ICU monitoring a common practice for patients undergoing craniocerebral procedures (Laan et al., 2020). In numerous neurosurgical centers across China, individuals receiving general anesthesia for such operations are routinely admitted to intensive care units for specialized observation and management. Individuals undergoing cranial surgery frequently experience nausea and vomiting of varying severity, with these manifestations being particularly prevalent among those sustaining mild to moderate cerebral injuries. Paradoxically, patients with severe neurological impairment [Glasgow Coma Scale (GCS) ≤ 8] often exhibit attenuated emetic responses due to suppressed brainstem reflex arcs (Brain Trauma Foundation et al., 2007b). These divergent clinical manifestations underscore the necessity for tailored postoperative management strategies. Effective control of vomiting not only enhances patient comfort but also prevents secondary complications including aspiration pneumonia and intracranial pressure elevation, which are critical determinants of neurological recovery (Andrews and Hawthorn, 1988; Eberhart et al., 2007).

Ondansetron, a therapeutic agent targeting 5-hydroxytryptamine subtype 3 (5-HT3) receptors through selective antagonism (Borner et al., 2020), has maintained a well-established safety profile through over two decades of clinical implementation (Garsed et al., 2014), solidifying its position as the pharmacological mainstay for perioperative emesis management in surgical care (Lee et al., 2020). Its mechanism involves competitive inhibition of serotonin binding at vagal afferent terminals in the chemoreceptor trigger zone and gastrointestinal tract (Hesketh, 2008). Clinical evidence supports its efficacy in reducing postoperative nausea and vomiting (PONV) across diverse surgical populations, including abdominal surgery patients (Zhang et al., 2019) and gynecological operation cohorts (Abdulhussain, 2022). Additionally, the administration of ondansetron can significantly reduce the incidence of PONV in patients undergoing craniotomy (Ryu et al., 2014).

While ondansetron prophylaxis demonstrates significant efficacy in preventing PONV following cranial neurosurgical interventions, its neuroprotective implications — specifically regarding 30- and 90 days mortality indices—remain inadequately substantiated in contemporary neurocritical care pharmacovigilance studies. This knowledge gap motivated our investigation into ondansetron’s impact on mortality outcomes in a targeted population of cranial surgery patients with GCS > 8. Our study employs propensity score matching (PSM) to address confounding factors inherent in retrospective analyses, aiming to provide evidence for optimized postoperative management protocols.

## 2 Materials and methods

### 2.1 Data source

The clinical data utilized in this investigation originated from the Medical Information Mart for Intensive Care IV (MIMIC-IV), an open-access critical care database jointly developed by Massachusetts Institute of Technology researchers and clinicians at Beth Israel Deaconess Medical Center (Johnson et al., 2023). This repository contains de-identified medical records spanning 2008–2022 from over 90,000 adult ICU admissions, incorporating multidimensional clinical parameters such as vital sign measurements, pharmacological interventions, laboratory biomarkers, and longitudinal outcome tracking. Authorized access was secured through completion of the Collaborative Institutional Training Initiative certification program (certification ID: 58746822), with principal investigator Dr. Guangdong Wang fulfilling all ethical and data protection requirements stipulated by the database custodians.

### 2.2 Participant selection

**FIGURE 1:**
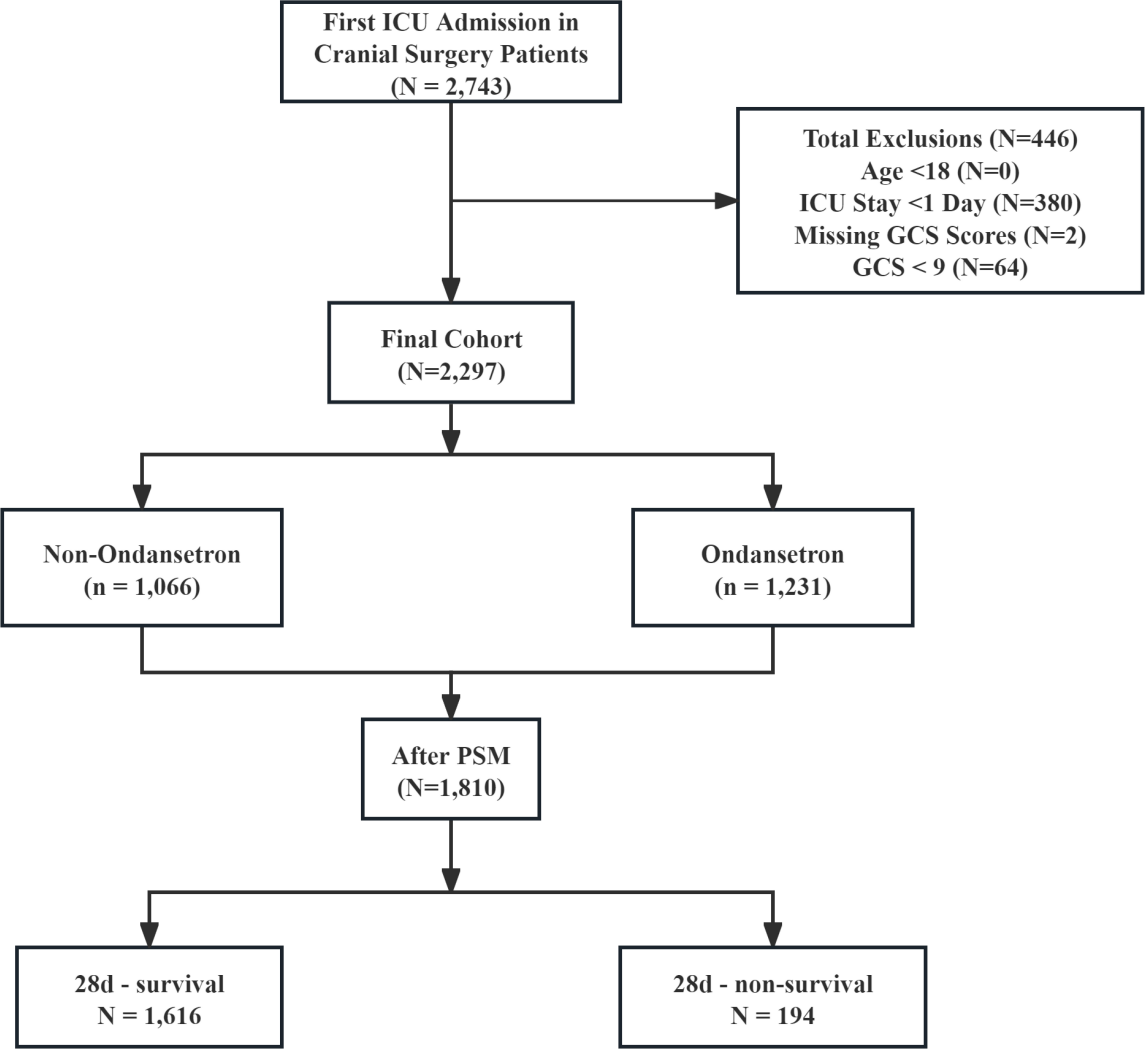
The flow chart of the study. GCS, Glasgow Coma Scale; ICU, intensive care unit; PSM, propensity-score matching.

The study population comprised patients undergoing neurosurgical interventions involving cerebral parenchyma, meninges, or ventricular systems, identified through ICD-9/10 coding. Eligible procedures encompassed diverse operative modalities including but not limited to craniotomy, lesion resection, shunt placement/revision, and intracranial device implantation, performed through open or percutaneous approaches across multiple neuroanatomical compartments. From an initial pool of 2,743 intensive care unit (ICU) cases, exclusion criteria eliminated individuals with age < 18 years (*n* = 0), admission duration < 24 h (*n* = 380), undocumented Glasgow Coma Scale (GCS) assessments (*n* = 2), and severe neurological impairment (GCS ≤ 8, *n* = 64). The resultant analytical cohort contained 2,297 subjects, stratified by ondansetron exposure status into 1,066 non-recipients and 1,231 recipients. PSM optimized cohort comparability, generating 905 matched dyads for final outcome evaluation (Figure 1).

### 2.3 Ondansetron exposure

Ondansetron exposure was defined as the administration of the medication via oral or intravenous routes within the first 24 h following ICU admission in cranial surgery patients, irrespective of dosage, formulation, or dosing frequency.

### 2.4 Variables

Baseline characteristics encompassed demographics (age, gender, race), physiological metrics (heart rate, systolic/diastolic blood pressure, respiratory rate, temperature, SpO2), and laboratory profiles (glucose, WBC, RBC, platelet, sodium, potassium, calcium, BUN, creatinine). Clinical assessments included Sequential Organ Failure Assessment (SOFA), GCS, and Charlson Comorbidity Index (CCI), with comorbidity documentation of myocardial infarction, congestive heart failure, chronic pulmonary disease, diabetes, hypertension, and malignancy. Therapeutic interventions analyzed epinephrine/norepinephrine use, mechanical ventilation (MV), continuous renal replacement therapy (CRRT), Ondansetron, and neuromuscular blockade. Outcome measures evaluated ICU/hospital length of stay and mortality rates (ICU/hospital/28 days/90 days) (Tables 1, 2).

**TABLE 1:** Baseline characteristics.

Variable	Before PSM					After PSM				
	Total (*n* = 2,297)	No (*n* = 1,066)	Yes (*n* = 1,231)	*P*	SMD	Total (*n* = 1,810)	No (*n* = 905)	Yes (*n* = 905)	*P*	SMD
Age (year)	62.82 (49.59, 73.37)	64.70 (52.65, 75.10)	61.03 (47.75, 71.93)	< 0.001	−0.17	63.54 (50.75, 73.71)	63.57 (50.54, 74.17)	63.41 (50.89, 73.41)	0.91	0.019
Gender, *n* (%)				< 0.001					0.89	
Female	1,058 (46.06)	449 (42.12)	609 (49.47)		0.147	801 (44.25)	399 (44.09)	402 (44.42)		0.007
Male	1,239 (53.94)	617 (57.88)	622 (50.53)		–0.15	1,009 (55.75)	506 (55.91)	503 (55.58)		–0.01
Race, *n* (%)				0.483					1	
Other	763 (33.22)	362 (33.96)	401 (32.58)		−0.03	602 (33.26)	301 (33.26)	301 (33.26)		0
White	1,534 (66.78)	704 (66.04)	830 (67.42)		0.03	1,208 (66.74)	604 (66.74)	604 (66.74)		0
Heart rate (bmp)	79.00 (68.00, 90.00)	79.00 (68.00, 90.75)	79.00 (68.00, 90.00)	0.902	−0.02	79.00 (68.00, 90.00)	79.00 (68.00, 90.00)	79.00 (68.00, 90.00)	0.78	0.001
SBP (mmHg)	133.00 (119.00, 147.00)	133.00 (119.00, 147.00)	133.00 (119.00, 146.00)	0.885	0.003	133.00 (119.00, 147.00)	133.00 (119.00, 148.00)	133.00 (119.00, 147.00)	0.7	−0.01
DBP (mmHg)	69.00 (60.00, 78.00)	68.00 (60.00, 79.00)	69.00 (60.00, 78.00)	0.995	0.001	69.00 (60.00, 78.00)	69.00 (60.00, 79.00)	69.00 (60.00, 78.00)	0.34	-0.03
Respiratory rate (bmp)	17.00 (14.00, 20.00)	17.00 (14.00, 20.00)	16.00 (14.00, 20.00)	0.008	−0.12	17.00 (14.00, 20.00)	17.00 (14.00, 20.00)	17.00 (14.00, 20.00)	0.88	−0.02
Temperature (°C)	36.72 (36.44, 37.00)	36.72 (36.39, 37.06)	36.67 (36.44, 37.00)	0.614	0.019	36.67 (36.44, 37.00)	36.67 (36.39, 37.06)	36.67 (36.44, 37.00)	0.75	0.023
Spo_2_ (%)	99.00 (97.00, 100.00)	99.00 (97.00, 100.00)	99.00 (97.00, 100.00)	0.04	−0.06	99.00 (97.00, 100.00)	99.00 (97.00, 100.00)	99.00 (97.00, 100.00)	0.43	−0.01
Glucose (mg/dL)	140.00 (117.00, 172.00)	138.00 (113.00, 174.00)	141.00 (120.00, 171.00)	0.13	−0.04	139.00 (116.00, 171.00)	136.00 (113.00, 171.00)	140.00 (119.00, 171.00)	0.09	0.017
SOFA	0.00 (0.00, 1.00)	1.00 (0.00, 1.00)	0.00 (0.00, 1.00)	0.017	−0.18	0.00 (0.00, 1.00)	0.00 (0.00, 1.00)	0.00 (0.00, 1.00)	0.82	−0.05
GCS	15.00 (14.00, 15.00)	15.00 (15.00, 15.00)	15.00 (14.00, 15.00)	< 0.001	−0.09	15.00 (14.00, 15.00)	15.00 (15.00, 15.00)	15.00 (14.00, 15.00)	0.07	−0.02
CCI	4.00 (2.00, 6.00)	4.00 (2.00, 7.00)	3.00 (1.00, 6.00)	< 0.001	−0.26	4.00 (2.00, 6.00)	4.00 (2.00, 6.00)	4.00 (2.00, 6.00)	0.54	0.029
RBC (10^9^/L)	3.98 (3.55, 4.39)	3.94 (3.49, 4.36)	4.01 (3.61, 4.41)	0.006	0.117	3.99 (3.55, 4.38)	3.97 (3.52, 4.39)	4.00 (3.58, 4.38)	0.47	0.028
WBC (10^9^/L)	12.30 (9.20, 15.60)	12.10 (9.10, 15.40)	12.40 (9.40, 15.80)	0.188	0.023	12.20 (9.03, 15.50)	12.20 (9.10, 15.40)	12.20 (8.90, 15.70)	0.93	−0
Platelet (10^9^/L)	217.00 (174.00, 269.00)	214.50 (168.25, 272.75)	217.00 (177.00, 265.50)	0.311	−0.02	215.00 (173.25, 267.75)	213.00 (169.00, 269.00)	217.00 (176.00, 265.00)	0.4	0.028
Sodium (mmol/L)	140.00 (137.00, 142.00)	140.00 (137.00, 142.00)	140.00 (137.00, 142.00)	0.753	0.027	140.00 (137.00, 142.00)	140.00 (137.00, 142.00)	140.00 (137.00, 142.00)	0.93	−0
Potassium (mmol/L)	3.90 (3.70, 4.20)	3.90 (3.60, 4.27)	3.90 (3.70, 4.20)	0.698	−0.05	3.90 (3.60, 4.30)	3.90 (3.60, 4.20)	3.90 (3.70, 4.30)	0.6	−0.06
Calcium (mg/dL)	8.50 (8.10, 8.90)	8.50 (8.10, 8.90)	8.50 (8.20, 8.90)	0.402	0.045	8.50 (8.10, 8.90)	8.50 (8.10, 8.90)	8.50 (8.10, 8.90)	0.98	0.011
BUN (mg/dL)	14.00 (10.00, 19.00)	15.00 (11.00, 20.00)	14.00 (10.00, 18.00)	0.001	−0.17	14.00 (11.00, 19.00)	14.00 (10.00, 19.00)	14.00 (11.00, 19.00)	0.5	−0.02
Creatinine (mg/dL)	0.80 (0.70, 1.00)	0.80 (0.70, 1.00)	0.80 (0.70, 1.00)	0.052	−0.09	0.80 (0.70, 1.00)	0.80 (0.70, 1.00)	0.80 (0.70, 1.00)	0.43	−0.01
Myocardial infarct, *n* (%)				0.065					0.58	
No	2,180 (94.91)	1,002 (94.00)	1,178 (95.69)		0.084	1725 (95.3)	865 (95.58)	860 (95.03)		–0.03
Yes	117 (5.09)	64 (6.00)	53 (4.31)		–0.08	85 (4.7)	40 (4.42)	45 (4.97)		0.025
Congestive heart failure, *n* (%)				< 0.001					0.55	
No	2,150 (93.6)	978 (91.74)	1,172 (95.21)		0.162	1,702 (94.03)	848 (93.70)	854 (94.36)		0.029
Yes	147 (6.4)	88 (8.26)	59 (4.79)		−0.16	108 (5.97)	57 (6.30)	51 (5.64)		−0.03
Chronic pulmonary disease, *n* (%)				0.949					0.89	
No	2,005 (87.29)	931 (87.34)	1,074 (87.25)		–0	1,588 (87.73)	793 (87.62)	795 (87.85)		0.007
Yes	292 (12.71)	135 (12.66)	157 (12.75)		0.003	222 (12.27)	112 (12.38)	110 (12.15)		–0.01
Diabetes, *n* (%)				0.044					0.46	
No	1,901 (82.76)	864 (81.05)	1,037 (84.24)		0.088	1,496 (82.65)	754 (83.31)	742 (81.99)		–0.04
Yes	396 (17.24)	202 (18.95)	194 (15.76)		–0.09	314 (17.35)	151 (16.69)	163 (18.01)		0.035
Malignant cancer, *n* (%)				0.425					0.86	
No	1,850 (80.54)	851 (79.83)	999 (81.15)		0.034	1437 (79.39)	717 (79.23)	720 (79.56)		0.008
Yes	447 (19.46)	215 (20.17)	232 (18.85)		–0.03	373 (20.61)	188 (20.77)	185 (20.44)		–0.01
Hypertension, *n* (%)				< 0.001					0.64	
No	1,049 (45.67)	438 (41.09)	611 (49.63)		0.171	794 (43.87)	402 (44.42)	392 (43.31)		–0.02
Yes	1,248 (54.33)	628 (58.91)	620 (50.37)		–0.17	1,016 (56.13)	503 (55.58)	513 (56.69)		0.022
Epinephrine, *n* (%)				0.585					1	
No	2,287 (99.56)	1,060 (99.44)	1,227 (99.68)		0.042	1,803 (99.61)	901 (99.56)	902 (99.67)		0.019
Yes	10 (0.44)	6 (0.56)	4 (0.32)		–0.04	7 (0.39)	4 (0.44)	3 (0.33)		–0.02
Norepinephrine, *n* (%)				< 0.001					0.35	
No	2,137 (93.03)	970 (90.99)	1,167 (94.80)		0.171	1,688 (93.26)	839 (92.71)	849 (93.81)		0.046
Yes	160 (6.97)	96 (9.01)	64 (5.20)		–0.17	122 (6.74)	66 (7.29)	56 (6.19)		–0.05
Neuroblock, *n* (%)				0.031					0.68	
No	2,263 (98.52)	1,044 (97.94)	1,219 (99.03)		0.111	1,786 (98.67)	892 (98.56)	894 (98.78)		0.02
Yes	34 (1.48)	22 (2.06)	12 (0.97)		–0.11	24 (1.33)	13 (1.44)	11 (1.22)		–0.02
MV, *n* (%)				< 0.001					0.96	
No	737 (32.09)	296 (27.77)	441 (35.82)		0.168	543 (30)	272 (30.06)	271 (29.94)		–0
Yes	1,560 (67.91)	770 (72.23)	790 (64.18)		–0.17	1267 (70)	633 (69.94)	634 (70.06)		0.002
CRRT, *n* (%)				0.003					1	
No	2,280 (99.26)	1,052 (98.69)	1,228 (99.76)		0.217	1,808 (99.89)	904 (99.89)	904 (99.89)		0
Yes	17 (0.74)	14 (1.31)	3 (0.24)		–0.22	2 (0.11)	1 (0.11)	1 (0.11)		0

SOFA, Sequential Organ Failure Assessment; GCS, Glasgow Coma Scale; OASIS, Oxford Acute Severity of Illness Score; CCI: Charlson Comorbidity Index; SpO_2_, oxygen saturation; SBP, systolic blood pressure; DBP, diastolic blood pressure; WBC, white blood cell count; RBC, red blood cell count; Platelet, platelet count; MV, mechanical ventilation; CRRT, continuous renal replacement therapy.

**TABLE 2:** Baseline characteristics after propensity-score matching (PSM) and 28 days survival vs. non-survival outcomes.

Characteristic	Overall (*n* = 1,810)	28 days survival (*n* = 1,616)	28 days no-survival (*n* = 194)	*P*-value
Age (year)	63.54 (50.75, 73.71)	62.75 (50.10, 72.79)	70.90 (58.38, 79.71)	< 0.001
Gender, *n* (%)				0.743
Female	801 (44%)	713 (44%)	88 (45%)	
Male	1,009 (56%)	903 (56%)	106 (55%)	
Race, *n* (%)				< 0.001
Other	602 (33%)	504 (31%)	98 (51%)	
White	1,208 (67%)	1,112 (69%)	96 (49%)	
Heart rate (bmp)	79.00 (68.00, 90.00)	79.00 (68.00, 90.00)	79.00 (67.00, 93.00)	0.485
SBP (mmHg)	133.00 (119.00, 147.00)	133.00 (119.00, 147.00)	132.50 (115.00, 154.00)	0.55
DBP (mmHg)	69.00 (60.00, 78.00)	69.00 (60.00, 78.00)	68.00 (60.00, 79.00)	0.926
Respiratory rate (bmp)	17.00 (14.00, 20.00)	16.00 (14.00, 20.00)	18.00 (16.00, 22.00)	< 0.001
Temperature (°C)	36.67 (36.44, 37.00)	36.72 (36.44, 37.00)	36.61 (36.33, 37.11)	0.242
Spo_2_ (%)	99.00 (97.00, 100.00)	99.00 (97.00, 100.00)	100.00 (98.00, 100.00)	< 0.001
Glucose (mg/dL)	139.00 (116.00, 171.00)	138.00 (116.00, 169.00)	149.00 (123.00, 193.00)	< 0.001
SOFA	0.00 (0.00, 1.00)	0.00 (0.00, 1.00)	1.00 (0.00, 2.00)	< 0.001
GCS	15.00 (14.00, 15.00)	15.00 (14.00, 15.00)	15.00 (15.00, 15.00)	0.004
CCI	4.00 (2.00, 6.00)	4.00 (2.00, 6.00)	5.00 (3.00, 7.00)	< 0.001
Myocardial infarct, *n* (%)	85 (5%)	77 (5%)	8 (4%)	0.69
Congestive heart failure, *n* (%)	108 (6%)	89 (6%)	19 (10%)	0.017
Chronic pulmonary disease, *n* (%)	222 (12%)	196 (12%)	26 (13%)	0.609
Diabetes, *n* (%)	314 (17%)	263 (16%)	51 (26%)	< 0.001
Malignant cancer, *n* (%)	373 (21%)	359 (22%)	14 (7%)	< 0.001
Hypertension, *n* (%)	1,016 (56%)	883 (55%)	133 (69%)	< 0.001
RBC (10^9^/L)	3.99 (3.55, 4.38)	4.01 (3.58, 4.39)	3.80 (3.26, 4.22)	< 0.001
WBC (10^9^/L)	12.20 (9.00, 15.50)	12.20 (8.95, 15.45)	12.05 (9.50, 16.20)	0.307
Platelet (10^9^/L)	215.00 (173.00, 268.00)	217.00 (177.00, 269.00)	192.00 (143.00, 250.00)	< 0.001
Sodium (mmol/L)	140.00 (137.00, 142.00)	140.00 (137.00, 142.00)	139.00 (136.00, 142.00)	0.244
Potassium (mmol/L)	3.90 (3.60, 4.30)	3.90 (3.70, 4.20)	3.90 (3.50, 4.40)	0.546
Calcium (mg/dL)	8.50 (8.10, 8.90)	8.50 (8.10, 8.90)	8.50 (8.00, 8.90)	0.61
BUN (mg/dL)	14.00 (11.00, 19.00)	14.00 (10.00, 19.00)	16.50 (12.00, 23.00)	< 0.001
Creatinine (mg/dL)	0.80 (0.70, 1.00)	0.80 (0.70, 1.00)	0.90 (0.70, 1.10)	< 0.001
Epinephrine, *n* (%)	7 (0%)	5 (0%)	2 (1%)	0.168
Norepinephrine, *n* (%)	122 (7%)	84 (5%)	38 (20%)	< 0.001
Neuroblock, *n* (%)	24 (1%)	21 (1%)	3 (2%)	0.737
MV, *n* (%)	1,267 (70%)	1,107 (69%)	160 (82%)	< 0.001
CRRT, *n* (%)	2 (0%)	1 (0%)	1 (1%)	0.203
Ondansetron, *n* (%)	905 (50%)	825 (51%)	80 (41%)	0.01
Los hospital (day)	7.68 (4.24, 15.78)	7.73 (4.32, 16.32)	7.13 (3.25, 13.93)	0.005
Hospital mortality, *n* (%)	161 (9%)	9 (1%)	152 (78%)	< 0.001
Los ICU (day)	3.08 (1.77, 7.39)	2.99 (1.73, 6.90)	5.13 (2.61, 9.76)	< 0.001
ICU mortality, *n* (%)	111 (6%)	1 (0%)	110 (57%)	< 0.001
90 days hospital mortality, *n* (%)	274 (15%)	80 (5%)	194 (100%)	< 0.001

SOFA, Sequential Organ Failure Assessment; GCS, Glasgow Coma Scale; OASIS, Oxford Acute Severity of Illness Score; CCI, Charlson Comorbidity Index; SpO_2_, oxygen saturation; SBP, systolic blood pressure; DBP, diastolic blood pressure; WBC, white blood cell count; RBC, red blood cell count, Platelet, platelet count; MV, mechanical ventilation, CRRT, continuous renal replacement therapy.

### 2.5 Outcomes

The study evaluated 28 days all-cause survival rates as the principal endpoint, with 90 days all-cause mortality constituting a secondary analytical focus.

### 2.6 Statistical analysis

The analytical cohort incorporated all enrolled subjects. Quantitative parameters followed distribution-dependent presentation: Gaussian-distributed metrics as mean ± SD, non-normal variables as median (IQR). Categorical intergroup comparisons implemented χ^2^ contingency testing, while continuous measures utilized parametric *t*-tests or Wilcoxon rank-sum evaluations based on normality verification.

PSM applied nearest-neighbor matching (1:1 ratio) with 0.05 caliper restriction. Covariate balance validation employed standardized covariate differences (threshold < 0.10). Survival relationships were quantified through multivariate Cox proportional hazards models and time-to-event analysis with Kaplan-Meier estimators (28/90 days endpoints).

The analyzed parameters demonstrated 95%+ data completeness (Supplementary Table 1). Residual absent values underwent multivariate imputation via chained equations (MICE) using R’s computational environment. Analytical computations were performed using R v4.4.1, with statistical significance determined at α = 0.05.

## 3 Results

### 3.1 Basic characteristics

**FIGURE 2:**
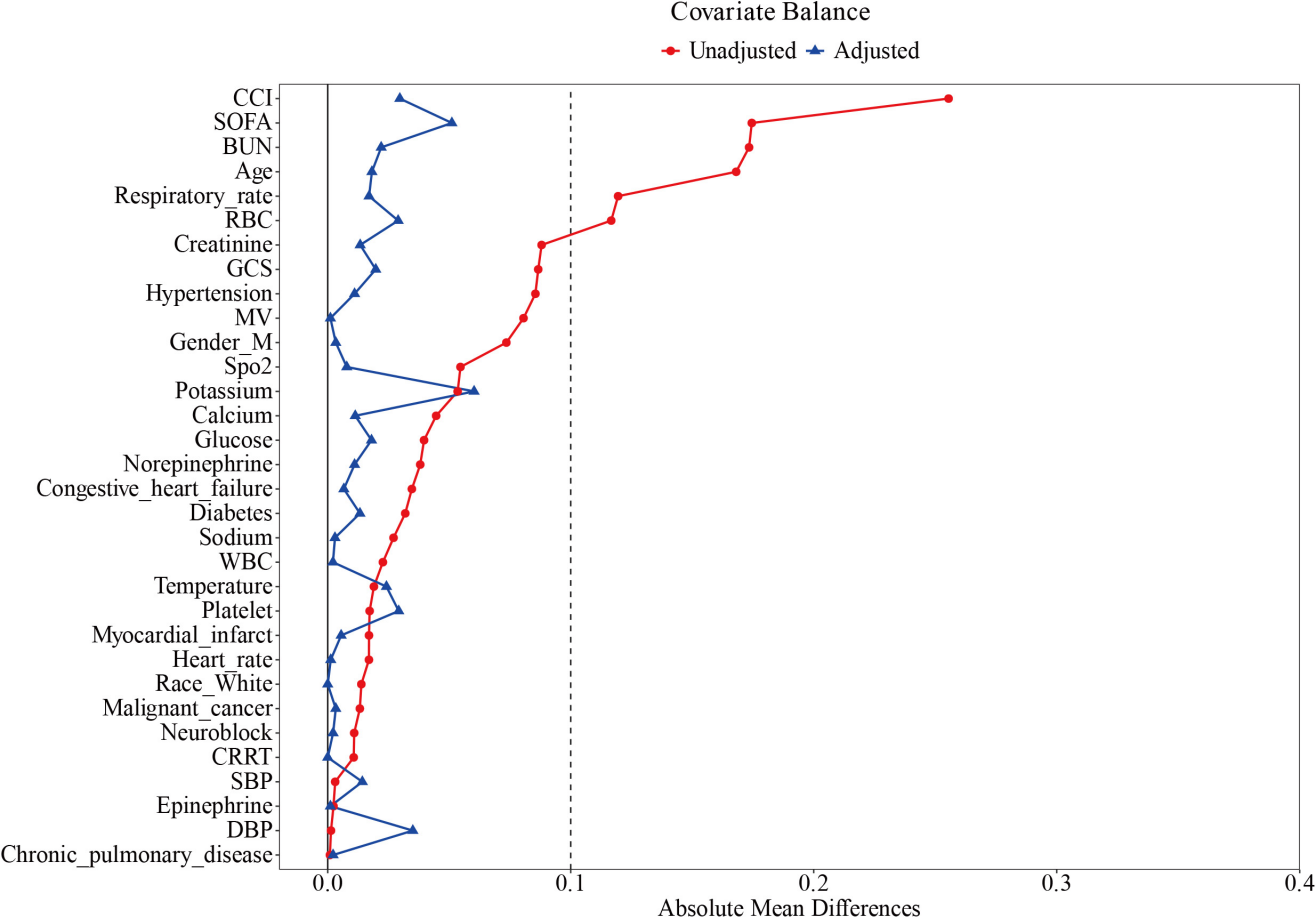
Standardized mean difference of variables before and after propensity score matching. SOFA, Sequential Organ Failure Assessment; GCS, Glasgow Coma Scale; CCI, Charlson Comorbidity Index; MV, mechanical ventilation; CRRT, continuous renal replacement therapy; WBC, white blood cells; SpO_2_, blood oxygen saturation; SBP, systolic blood pressure; DBP, diastolic blood pressure.

The pre-PSM comprised 2,297 subjects stratified into 1,231 ondansetron recipients and 1,066 non-recipients. Significant intergroup disparities emerged across demographics (age and gender), respiratory physiology (respiratory rate), clinical severity scores (SOFA, GCS, and CCI), laboratory profiles (RBC, platelet and BUN), comorbid conditions (diabetes, hypertension, congestive heart failure), and therapeutic interventions (norepinephrine administration, neuromuscular blockade, MV, and CRRT), with all comparative analyses reaching statistical significance (*P* < 0.05). PSM yielded 905 matched pairs, achieving balanced baseline characteristics across all measured covariates (Figure 2 and Table 1).

Analysis of the propensity-matched cohort (*n* = 1,810) identified statistically significant associations between 28 days mortality and multiple covariates (*P* < 0.05), including demographic characteristics (age, ethnicity), physiological metrics (respiratory rate, SpO2, glucose levels), clinical severity scores (SOFA, GCS, and CCI), comorbidities (congestive heart failure, diabetes mellitus, malignancy, hypertension), hematologic parameters (RBC and platelet count, BUN, creatinine), therapeutic interventions (norepinephrine, ondansetron and MV) (Table 2). Potential prognostic variables underwent univariate Cox proportional hazards screening (Supplementary Table 2) to identify candidate predictors of postoperative prognosis.

### 3.2 Primary outcome

**FIGURE 3:**
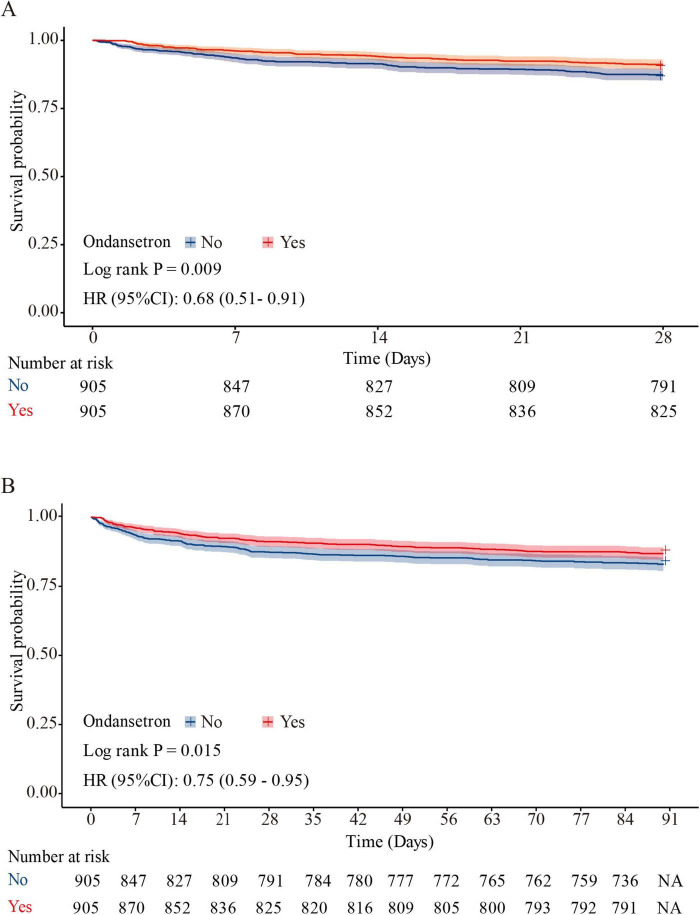
Kaplan–Meier survival curve for 28 and 90 days mortality. Ondansetron use was associated with improved 28 days survival (HR = 0.68, 95% CI: 0.51–0.91; log-rank *P* = 0.009) **(A)**, and 90 days survival (HR = 0.75, 95% CI: 0.59–0.95; *P* = 0.015) **(B)** in the matched cohort.

Baseline characteristics impacting 28 days survival (Table 2) informed the selection of clinically relevant parameters for univariate Cox hazard modeling, with variable prioritization guided through iterative consultation with neurosurgical specialists (Supplementary Table 2). Multivariable Cox proportional hazards regression of significant predictors (Table 3, Model 3) revealed ondansetron’s protective association with reduced 28 days mortality risk (HR = 0.69, 95% CI: 0.52–0.92; *P* = 0.012). Kaplan-Meier survival curves substantiated this therapeutic benefit in the neurocritical care context (HR = 0.68, 95% CI: 0.51–0.91; log-rank *P* = 0.009) (Figure 3A).

**TABLE 3:** Association between ondansetron use and mortality in cranial surgery patients after propensity-score matching (PSM).

Variables	Model 1		Model 2		Model 3	
	HR (95% CI)	*P*	HR (95% CI)	*P*	HR (95% CI)	*P*
**28 days mortality**						
**Ondansetron**						
No	1.00 (reference)		1.00 (reference)		1.00 (reference)	
Yes	0.68 (0.51, 0.91)	0.009	0.68 (0.51, 0.90)	0.007	0.69 (0.52, 0.92)	0.012
**90 days mortality**						
**Ondansetron**						
No	1.00 (reference)		1.00 (reference)		1.00 (reference)	**–**
Yes	0.74 (0.59, 0.95)	0.015	0.74 (0.58, 0.94)	0.014	0.74 (0.58, 0.94)	0.014

HR, hazard ratio; CI, confidence interval. Model 1: crude. Model 2: adjust: age, gender, race. Model 3: adjust: age, gender, race, congestive heart failure, diabetes, malignant cancer, hypertension, respiratory rate, Spo_2_, glucose, platelet, BUN, Sequential Organ Failure Assessment (SOFA), Glasgow Coma Scale (GCS), Charlson Comorbidity Index (CCI), norepinephrine, mechanical ventilation (MV).

### 3.3 Secondary outcomes

Multivariable Cox proportional hazards regression (Table 3, Model 3) demonstrated a significant inverse correlation between ondansetron exposure and 90 days mortality (HR = 0.74, 95% CI: 0.58–0.94; *P* = 0.014). Survival trajectories analyzed via Kaplan-Meier methodology during the 90 days observation period corroborated ondansetron’s sustained therapeutic benefit in neurocritical care populations (HR = 0.75, 95% CI: 0.59–0.95; *P* = 0.015) (Figure 3B).

### 3.4 Subgroup analysis

**FIGURE 4:**
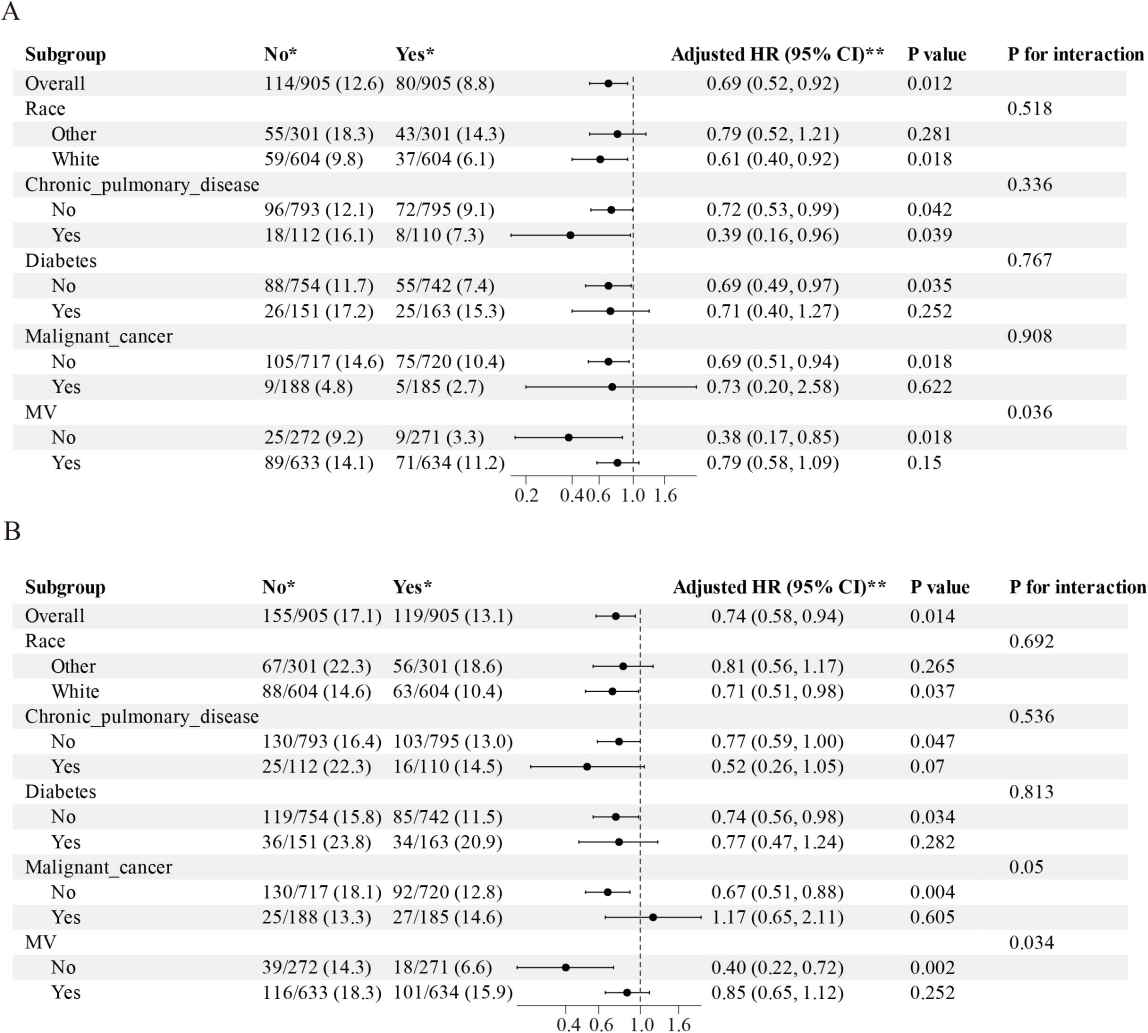
The association between ondansetron administration and short-term mortality in various subgroups. **(A)** The relationship between ondansetron use and 28 days mortality within these subgroups, and **(B)** the association between ondansetron use and 90 days mortality across the identical subgroup. HR, hazard ratios; 95% CI, confidence intervals; MV, mechanical ventilation.

Subgroup evaluations revealed consistent mortality risk reduction with ondansetron administration across demographic and clinical strata. Adjusted analyses demonstrated significant 28 days survival benefits (aHR = 0.69, 95% CI: 0.52–0.92; *P* = 0.012) and 90 days protective effects (aHR = 0.74, 95% CI: 0.58–0.94; *P* = 0.014), particularly pronounced in White ethnicity cohorts, non-diabetic patients, those without malignancies, and individuals not requiring mechanical ventilation (all aHR < 1, *P* < 0.05). Interaction testing showed non-significant effect modifications for ethnicity, chronic pulmonary disease, diabetes, and malignancy subgroups (*P* interaction > 0.05), while mechanical ventilation status exhibited significant interaction (*P* interaction < 0.05), with therapeutic consistency observed at both 28- and 90 days intervals (Figures 4A, B).

## 4 Discussion

This retrospective propensity - matched study evaluated the impact of ondansetron on clinical outcomes in cranial surgery patients. The findings suggest that ondansetron use is associated with a significant reduction in 28 and 90 days mortality in this patient population.

The observed mortality reduction is a notable finding, considering the complex postoperative course of cranial surgery patients. The mechanism through which ondansetron exerts this effect might be multi - faceted. As a central nervous System 5 - HT3 receptor antagonist (Kirchhoff et al., 2019), its well-established antiemetic property could play a role (Basak et al., 2019; Mascarenhas et al., 2019; Vaid et al., 2020). Ondansetron exhibits high selectivity as a 5-HT3 receptor antagonist, demonstrating 250- to 500-fold greater binding affinity for 5-HT3 receptors compared to 5-HT1A and 5-HT2B subtypes (Wang et al., 2019). The 5-HT3 receptor subtype demonstrates anatomical localization within peripheral vagal afferent terminals and dense concentration in the nucleus tractus solitarii of the brainstem’s emetic circuitry (Tyers, 1992). The high selectivity for 5-HT3 receptors and lack of antagonistic activity toward dopamine and other non-5-HT3 receptors result in ondansetron’s minimal neurological side effects and low incidence of extrapyramidal symptoms (Jueckstock et al., 2010; Tyers, 1992; Yin et al., 2020). By reducing PONV, ondansetron may prevent secondary complications such as aspiration pneumonia and intracranial pressure elevation (Andrews and Hawthorn, 1988; Eberhart et al., 2007). Aspiration pneumonia is a common and potentially life - threatening complication in hospitalized patients (Ko et al., 2010; Thomson et al., 2016; Xu et al., 2015), and acute intracranial hypertension is a common and life-threatening neurological complication often directly related to poor prognosis of patients (Vaibhav et al., 2020). Clinical studies further demonstrate that aspiration pneumonia in neurosurgical and critically ill patients is associated with increased mortality (Thomson et al., 2016; Xu et al., 2015), while acute intracranial hypertension has long been recognized as a major determinant of poor neurological outcomes and death (Brain Trauma Foundation, 2007a). These findings strengthen the clinical plausibility of our proposed pathway, whereby ondansetron reduces PONV, thereby lowering the incidence of aspiration events and ICP crises, ultimately contributing to improved survival in cranial surgery patients. Moreover, recurrent nausea and vomiting may precipitate acute hypertensive episodes, thereby elevating the risk of intraoperative intracranial hemorrhage. Severe intracranial hemorrhagic events may precipitate cerebral herniation through mass effect displacement (Crash-3 trial collaborators, 2019), culminating in catastrophic neurological deterioration secondary to brainstem compression and irreversible parenchymal injury (Gallagher et al., 2018; Song et al., 2017). In cranial surgery patients, the administration of ondansetron may be associated with maintenance of intracranial pressure stability, prevention of aspiration pneumonia, and preservation of hemodynamic equilibrium constitute critical perioperative priorities. The systematic mitigation of these interrelated risk factors may underlie its beneficial effects on improving patient outcomes.

Moreover, emerging evidence of ondansetron’s potential neuroprotective effects through modulation of inflammatory cascades and oxidative stress pathways may also be relevant (Hassan et al., 2024; Tian et al., 2023). Although these mechanisms remain underexplored in the neurosurgical context, they could offer additional explanations for the reduced mortality observed in our study. Inflammation and oxidative stress are known to play important roles in the pathophysiology of brain injury and recovery (Chen et al., 2022; Magid-Bernstein et al., 2022). Ondansetron might be interfering with these processes, leading to better outcomes. Recent studies provide more direct evidence supporting these hypotheses. Network pharmacology and experimental models have shown that ondansetron suppresses neutrophil extracellular trap (NET) formation by downregulating neutrophil elastase, MPO, and PAD4, while reducing pro-inflammatory cytokines such as IL-6, IL-1β, and TNF-α (Tao et al., 2024). Animal studies further suggest that ondansetron mitigates blood–brain barrier disruption, brain edema, and astrocytic activation, and confers anti-apoptotic effects by decreasing Bax and calcineurin expression while increasing Bcl-2 levels (Sharma et al., 2019). Nevertheless, these explanations remain largely hypothetical, and future mechanistic experiments and prospective clinical studies are required to confirm whether these pathways play a causal role in the improved outcomes observed in cranial surgery patients.

In the subgroup analysis, a significant interaction was observed between MV status and the effect of ondansetron on both 28 and 90 days mortality. Specifically, ondansetron use was associated with a marked reduction in short-term mortality among patients who did not require MV (28 days HR = 0.38, 95% CI: 0.17–0.85; 90 days HR = 0.40, 95% CI: 0.22–0.72), whereas the survival benefit was attenuated and no longer statistically significant in patients receiving MV. This finding may reflect underlying differences in baseline severity and clinical trajectory. Patients requiring MV generally present with more severe neurological impairment, hemodynamic instability, and a higher burden of complications such as ventilator-associated pneumonia, prolonged sedation, and secondary infections, which may diminish or overshadow the potential protective effects of ondansetron. By contrast, in non-ventilated patients with relatively preserved physiological reserve, the ability of ondansetron to mitigate aspiration events, stabilize intracranial pressure, and reduce perioperative complications could translate into a more pronounced survival benefit. These observations suggest that the therapeutic impact of ondansetron may be more evident in less severely ill patients, and highlight the need for future prospective studies to clarify whether MV status is a true effect modifier or primarily a marker of confounding severity.

There are several limitations to this study. Firstly, as a retrospective study, it is subject to inherent biases despite the use of propensity score matching. Residual confounding may still exist, as it is difficult to account for all potential confounders in a retrospective design. Secondly, the data source is from a single database (MIMIC-IV), which may limit the generalizability of the results. Different patient populations in other regions or hospitals may have different characteristics and responses to ondansetron. Thirdly, the dosage, timing, and duration of ondansetron administration were not standardized in the dataset, which may have influenced the observed associations. Fourthly, important perioperative variables such as the type of surgery, duration of the procedure, and intraoperative blood loss were not recorded, and these unmeasured factors could potentially confound the outcomes. Finally, the study only evaluated short-term mortality (28 and 90 days), and long-term outcomes such as neurological function recovery and quality of life were not assessed.

## 5 Conclusion

This study provides evidence that ondansetron may be beneficial in reducing short - term mortality in cranial surgery patients with GCS > 8. However, future prospective studies with larger and more diverse patient populations are needed to confirm these findings, explore the underlying mechanisms further, and evaluate the long - term impact of ondansetron on patient outcomes. This could help in developing more optimized postoperative management protocols for cranial surgery patients.

## Data Availability

The datasets presented in this study can be found in online repositories. The names of the repository/repositories and accession number(s) can be found in the article/Supplementary material.
