# Supplementary material for: The impact of ondansetron on clinical outcomes in cranial surgery patients: a propensity-matched analysis of retrospective data
Source: Front Aging Neurosci. 2025 Sep 23;17:1627353. doi: 10.3389/fnagi.2025.1627353 (PMC12500680; doi:10.3389/fnagi.2025.1627353)
Supplement: Supplementary file 1 [file Table_1.docx]

| **Table S1 Missing Variables and Their Percentages** | |
| --- | --- |
| Variable | Missing Value（%） |
| Calcium | 3.83108402 |
| RBC | 1.65433174 |
| BUN | 1.65433174 |
| WBC | 1.61079669 |
| Platelet | 1.61079669 |
| Potassium | 1.61079669 |
| Sodium | 1.56726165 |
| Creatinine | 1.56726165 |
| Respiratory rate | 0.4788855 |
| Glucose | 0.4788855 |
| WBC, white blood cells; RBC, red blood cells; BUN, blood urea nitrogen. |  |

| **Table S2 Univariable Cox Regression For 28-Day All-Cause Mortality** | | |
| --- | --- | --- |
| Variables | *P* | HR (95%CI) |
|  |  |  |
| Gender |  |  |
| F |  | 1.00 (Reference) |
| M | 0.803 | 0.96 (0.73, 1.28) |
| Race |  |  |
| Other |  | 1.00 (Reference) |
| White | <0.001 | 0.46 (0.35, 0.61) |
| Age | <0.001 | 1.02 (1.01, 1.03) |
| Respiratory rate | <0.001 | 1.07 (1.04, 1.09) |
| Spo2 | <0.001 | 1.19 (1.09, 1.29) |
| Glucose | <0.001 | 1.01 (1.01, 1.01) |
| SOFA | <0.001 | 1.33 (1.22, 1.46) |
| GCS | 0.112 | 1.13 (0.97, 1.31) |
| CCI | 0.002 | 1.07 (1.03, 1.12) |
| Congestive heart failure |  |  |
| No |  | 1.00 (Reference) |
| Yes | 0.016 | 1.79 (1.11, 2.87) |
| Diabetes |  |  |
| No |  | 1.00 (Reference) |
| Yes | <0.001 | 1.74 (1.27, 2.40) |
| Malignant cancer |  |  |
| No |  | 1.00 (Reference) |
| Yes | <0.001 | 0.28 (0.16, 0.49) |
| Hypertension |  |  |
| No |  | 1.00 (Reference) |
| Yes | <0.001 | 1.73 (1.28, 2.35) |
| WBC | 0.079 | 1.02 (1.00, 1.04) |
| Platelet | <0.001 | 0.99 (0.99, 0.99) |
| BUN | <0.001 | 1.03 (1.02, 1.04) |
| Norepinephrine |  |  |
| No |  | 1.00 (Reference) |
| Yes | <0.001 | 3.81 (2.67, 5.43) |
| MV |  |  |
| No |  | 1.00 (Reference) |
| Yes | <0.001 | 2.08 (1.44, 3.01) |
| SOFA, Sequential Organ Failure Assessment; GCS, Glasgow Coma Scale; CCI, Charlson Comorbidity Index; BUN, blood urea nitrogen; MV, mechanical ventilation. | | |
